# Maternal obesity is the new challenge; a qualitative study of health professionals’ views towards suitable care for pregnant women with a Body Mass Index (BMI) ≥30 kg/m^2^

**DOI:** 10.1186/1471-2393-12-157

**Published:** 2012-12-19

**Authors:** Debbie M Smith, Alison Cooke, Tina Lavender

**Affiliations:** 1 The School of Psychological Sciences, The University of Manchester, Oxford Road, Manchester, M13 9PL, UK; 2 The School of Nursing, Midwifery and Social work, The University of Manchester, Oxford Road, Manchester, M13 9PL, UK

**Keywords:** Maternal obesity, Health professionals, Qualitative research, Communication, Training needs

## Abstract

**Background:**

An increase in the number of women with maternal obesity (Body Mass Index [BMI] ≥30 kg/m^2^) has had a huge impact on the delivery of maternity services. As part of a programme of feasibility work to design an antenatal lifestyle programme for women with a BMI ≥30 kg/m^2^, the current study explored health professionals’ experiences of caring for women with a BMI ≥30 kg/m^2^ and their views of the proposed lifestyle programme.

**Method:**

Semi-structured interviews with 30 health professionals (including midwives, sonographers, anaesthetists and obstetricians) were conducted and analysed using thematic analysis. Recruitment occurred in two areas in the North West of England in early 2011.

**Results:**

Three themes were evident. Firstly, obesity was seen as *a conversation stopper*; obesity can be a challenge to discuss. Secondly, obesity was seen as *a maternity issue*; obesity has a direct impact on maternity care and therefore intervention is needed. Finally, the *long-term impact of maternal obesity intervention*; lifestyle advice in pregnancy has the potential to break the cyclic obesity relationship. The health professionals believed that antenatal lifestyle advice can play a key role in addressing the public health issue of obesity as pregnancy is a time of increased motivation for women with a BMI ≥30 kg/m^2^.

**Conclusions:**

Maternal obesity is a challenge and details of the training content required for health professionals to feel confident to approach the issue of maternal obesity with women are presented. Support for the antenatal lifestyle programme for women with a BMI ≥30 kg/m^2^ highlights the need for further exploration of the impact of interventions on health promotion.

## Background

Increasing rates of maternal obesity (Body Mass Index (BMI) ≥30 kg/m^2^) are a cause for concern for health professionals in clinical practice due to the increased risk for maternal complications [1,2], fetal complications [3] and the negative impact on their clinical time and resources [4,5]. Data suggests that maternal obesity is increasing in the USA [6] and the UK [7].

Clinical guidelines in the UK recommend that advice and support should be provided in early pregnancy to encourage a healthy pregnancy for mother and baby [8-11]. The role of healthy eating and physical activity in the prevention of gestational diabetes and excessive weight gain must be explained to women [9]. However, there is currently little evidence to inform the content and structure of antenatal weight management or health lifestyle interventions [12,13]. Recent literature reviews have concluded that dietary and lifestyle interventions have a role in the reduction of; postnatal weight retention, the incidence of gestational diabetes and excessive weight gain [14,15]. However, due to the quality of studies results must be interpreted carefully and more research conducted [16].

A meta-synthesis exploring the maternity experience for women with a BMI ≥30 kg/m^2^ found that when pregnant, women demonstrated understanding of the benefits of a healthy lifestyle and also wanted to receive healthy lifestyle advice so that they could lose weight in the postnatal stage [17]. Currently, little is known about the implementation of recent guidelines in clinical practice and whether the needs of women with a BMI ≥30 kg/m^2^ are met. To fully understand which intervention is most suitable with this target group, the views of health professionals need to be explored as they will act as gate-keepers and refer women to interventions or be a part of the teams delivering the interventions. Both involve health professionals feeling able to discuss the issue of weight with women.

Several recent small scale qualitative studies suggest numerous barriers to discussing weight with women and thus implementing maternal obesity guidelines. For example, an English study found that midwives consider lifestyle and obesity as low priority in maternity care [18]. Another English study found that the midwives reported communication difficulties due to the sensitivity of the topic [19]. Also in another study, a lack of resources, finance and equipment were raised by health professionals as barriers to discussing weight management with women [20]. A study in Australia with midwives found that confidence in addressing weight was low when knowledge was low, something that can be provided with specific training [21]. The training needs of those working with maternal obesity and the need for a multi-disciplinary approach were also found in a study of community-based service providers [22]. Women perceived the lack of weight focused advice from health professionals during maternity care as an indication that the health professionals were not concerned about their weight [23]. Finally, one study calls for midwives to recognise obesity as a chronic disease and offer pregnant women with a BMI ≥30 kg/m^2^ treatment in the form of advice [24]. In order to do this, the currently held views and experiences of health professionals in the UK must be explored and understood.

The current exploratory study was conducted as part of a programme of work to design and evaluate an innovative community-based 10-week antenatal lifestyle course for women with a BMI ≥ 30 kg/m^2^ (The Lifestyle Course - TLC). Findings of the studies conducted in this feasibility stage will be used to inform and design formal studies of the TLC (following the MRC complex intervention framework, [25]). In summary, TLC was run and designed by a multi-disciplinary team (including midwives and a health psychologist). The group-based sessions were run in the community for one and a half hours a week for 10-weeks, the sessions underpinned by health psychology theory with behaviour change techniques used. A multi-centred feasibility study evaluating the suitability and acceptability of TLC for the women with a BMI ≥ 30 kg/m^2^ was run in conjunction with this study [25]. The aim of the current study was to understand the health professionals’ experiences and views towards TLC. In addition, the interviews aimed to examine health professionals’ views of the impact of TLC on their current clinical practice. The outcomes of this study are to provide information on the feasibility of community lifestyle interventions such as TLC. This study is part of a programme of work that involves assessing the feasibility of TLC for the target group (pregnant women with a BMI ≥ 30 kg/m^2^). Triangulation of data enables us to assess the validity of qualitative data. The views of the health professionals is essential to understand if maternal interventions are to be implemented. Within the clinical setting, they act as as gate keepers to interventions and services for women*.* In addition, these health professionals care for a large number of pregnant women with a BMI ≥ 30 kg/m^2^ and thus may have a clear and unique understanding of the clinical challenges of supporting this target group at a personal and clinical level. A range of health professionals who offer antenatal care to women with a BMI ≥30 kg/m^2^ were included in this study (e.g., midwives, sonographers, anaesthetists and obstetricians).

## Methodology

### Sample

A purposive sample was recruited for this exploratory study from two hospital sites where women were recruited for a multi-centred feasibility study in which they were invited to attend an antenatal lifestyle programme [26]. None of the health professionals recruited had been involved in the running of TLC. Both areas were situated in the North West of England, one of England’s most deprived regions in terms of health inequalities [27] and obesity [28]. A variety of health professionals within the standard maternity care framework were approached to ensure a holistic view was presented. Written consent was obtained from all participants before the interview commenced.

### Procedure

Ethical approval was received from the Local Research Ethics Committee (Ref: 09/H1003/80), Research & Development Departments and The University. A semi-structured interview approach allowed the health professionals to lead the flow of discussion using a topic guide based on previous literature and enabled an inductive approach to analysis to be taken. Based on previous experience of qualitative work with health professionals, the interviews were designed to be short in length (approximately 30 minutes). The topic guide was designed to address four topics to meet the study aims: weight management in pregnancy, physical activity in pregnancy, services to support the needs of pregnant women with a BMI ≥30 kg/m^2^ and other (including training needs). The interviews began by asking the interviewees about their current experience of caring for women with a BMI ≥30 kg/m^2^ (encompassing all four topics). Interviewees were then asked about their experience and views towards TLC (encompassing all four topics above). All interviews were conducted by one researcher in a private room, were audio-recorded and transcribed verbatim. It was anticipated that 40–50 interviews would be conducted. However, the interview data were discussed between two of the authors at several points during the recruitment process and it was agreed that data saturation was met after 30 interviews were conducted and thus recruitment ended.

### Analysis

Analysis was conducted by two researchers to reduce interpreter bias. Thematic analysis was used to interpret data without losing detail [29]. Transcripts were read independently several times and, following an inductive approach, highlighted issues that summarised the health professionals’ accounts (themes). These themes that summarised the interview data were discussed and collaboratively defined due to a high level of agreement between the two researchers. An audit trail was kept to ensure transparency in the analysis. The health professionals were treated as a homogenous group as they all contributed to the maternity care of the target group; therefore, no horizontal analysis was conducted.

## Results

Thirty-seven health professionals were approached; 30 were willing to be interviewed (81% response rate). The length of interviews ranged from 10 to 34 minutes. The sample was representative of most professionals involved with the maternity care of women with a BMI ≥30 kg/m^2^, including midwives, sonographers, anaesthetists and obstetricians. To maintain anonymity of individual health professionals, all reference to the number recruited from each hospital location and their profession have been removed and replaced by a participant code (Health Professional [HP] 1 - HP30).

Three main themes portrayed the health professionals’ experience of providing maternity care for women with a BMI ≥30 kg/m^2^ and the role of the antenatal lifestyle programme; *obesity as a conversation stopper*, *obesity as a maternity issue* and *the long-term impact of maternal obesity intervention*. Anonymous quotes from the 30 interviews will illustrate these themes and are displayed in Table 1. The interviewees’ views were cohesive supporting the lack of separation based on profession or recruitment hospital.

**Table 1:** Quotes from the 30 interviews with health professionals to illustrate the three themes and subsequent sub-themes highlighted in these interviews

**Theme**	**Subtheme**	**Example quote (participant code)**
Obesity as a conversation stopper		
	Perceptions of obesity and the impact on communication	*“…to try and broach the subject with some women, it can be difficult because they are vulnerable and obviously it can be embarrassing and hurtful…”* (HP17)
	Delivery of information	*“…you can give out patient information leaflets but whether they read them is another thing – they’ve got so many”* (HP10)
		*“…you don’t want to upset them by saying they’re overweight or fat and you don’t really know what words to use sometimes…”* (HP13)
	Lack of information	*“…we should be able to offer things for obese ladies but it’s about us knowing what there is out there and what we can signpost these ladies off to”* (HP5)
	Knowledge and/or need for training	*“When we first started tackling HIV, oh we can’t possibly do that…I mean now we do it all and it’s a matter of course and nobody even thinks about it”* (HP13)
		*“…we’re always striving to give better advice to pregnant women…it would be nice to have a bit more knowledge around you to be able to advise people that way…it’s knowing sometimes what’s the right advice to give…only by going and researching yourself…”* (HP19)
Obesity as a maternity issue		
	Intervention is needed	*“…they* [women they cared for who attended the antenatal lifestyle programme] *were quite positive about the programme…had learnt a lot about healthy eating…would actually continue with their lifestyle change even after the baby was born”* (HP3)
		*“I think it should be embedded in the health service but also across the community services. It should be a joint venture. And education. I think the opportunities are there everywhere for us all to be delivering on that package and it should be integral to all services so that it’s seen to be the norm … that’s how it becomes acceptable…”* (HP26)
	Continuous care	*“Whoever reviews them, the booking midwife…whoever reviews them in the…reviews like let it be obstetricians and midwives, all levels of doctors…then they go to the GP and the booking midwives, that should be a good start and that should be continued at every visit”* (HP11)
	Impact on clinical practice	*“…looking at it practically and realistically it’s just another role* [providing information about maternal obesity]*…that you’re expected to fill and I just don’t think practically on a day to day basis you would have time to do it…”* (HP18)
		*“Main issue with obesity is…shoulder problems…it hurts your shoulder and some people have back problems as well”* (HP6)
Long-term impact of maternal obesity intervention		*“Healthy eating plans, weight management programmes, exercise programmes…are relevant not just for pregnancy but pre-conceptually and postnatally so that women even if they’ve a problem in this pregnancy can improve their health and well-being for the next pregnancy. I think that’s really important we don’t…ignore what we can see in front of us, we do something about it”* (HP26)
		*“…the cycle has to be broken somewhere and it’s finding the right balance of where to break that circle and how to do it effectively”* (HP2)

### Theme 1 - Obesity as a conversation stopper

The associated difficulties of introducing obesity into conversation were discussed in great detail. Four sub themes give more insight; *perceptions of obesity and the impact on communication*, *delivery of information*, *lack of information and/or knowledge* and *the need for training*.

#### Perceptions of obesity and the impact on communication

Weight was referred to as a sensitive topic which some felt reluctant to introduce due to the risk of causing offence and feelings of embarrassment on their part. However, all of the health professionals felt that the risks needed to be discussed and, where the evidence-base outlining the possible risks could be provided, it was slightly easier to discuss.

#### Delivery of information

Many issues were discussed within this sub theme; information overload for the women, correct words to use, leaflets versus one-to-one approach and a lack of standard obesity questions. The early maternity care appointments were generally viewed as causing “…*information overload*” (HP20) for women. Health professionals felt responsible, as they have to address a large number of issues within a short time.

Health professionals felt that they *“…have to choose our words very carefully…”* (HP30) due to the sensitivity of obesity; in particular they mentioned the use or avoidance of certain words that may cause offence such as “..*morbidly obese…”* (HP10).

Leaflets were viewed as only helping the minority as women receive a large number and they work on the premise that they take responsibility for their own health so *“…motivation has to come from them…”* (HP22). Finally, some health professionals suggested it may be easier to broach the subject of obesity with women if there were standard questions in the maternity booking history or notes.

#### Lack of information and/or knowledge

A lack of information and/or knowledge about obesity on the part of both the health professional and the women were found. Health professionals suggested that they had a basic knowledge of the possible risks associated with obesity. This resulted in a lack of confidence to provide advice. In addition, the health professionals also reported a lack of knowledge about where to refer the women for tailored support. All of the health professionals expressed a desire to increase their knowledge about possible risks and available services.

Health professionals felt that there was a lack of awareness amongst women about the possible risks associated with obesity. They all felt that women should be given information about the possible risks so that they can make informed choices. One reason given for the women’s lack of knowledge was the lack of available information.

#### Need for training

Obesity was compared to other sensitive topics that, over time and with training, became routine without stigma, such as HIV and smoking. Training was discussed as being required in several forms; to increase the health professionals’ knowledge of maternal obesity, to develop skills to approach obesity in a sensitive manner and to increase their knowledge of specialised support services. Knowledge was limited to what health professionals had taught themselves. Finally, the health professionals valued the antenatal lifestyle programme offered as part of the current feasibility study as they were unaware of any other services that offered this support.

### Theme 2 - Obesity as a maternity issue

Three sub themes gave more detail as to why health professionals view obesity as a maternity issue; *intervention is needed*, *continuous care that starts early (preconception to postnatal)* and *impact on clinical practice*.

#### Intervention is needed

The health professionals commended the recently developed maternal obesity guidelines. All the health professionals felt that pregnancy was a good opportunity to intervene as women are *“…motivated…”* (HP14) and want *“…the best outcome …”* (HP26). The majority of the health professionals had heard of TLC but their knowledge of the study differed depending on whether they had cared for women who had attended or not. Four suggestions were made in reference to what is needed in maternity care to address obesity; the feasibility study had a positive impact on women, a lifestyle approach is needed, a group setting is best and a community location is required.

Health professionals reported that TLC (the programme that was designed specifically for the feasibility study running in conjunction with this study) was well received by women; many reported it having a positive impact on women to make permanent lifestyle changes. The health professionals reported the views of the women they cared for and also gave their own reflections of TLC in terms of added social support for these women. It was also a useful addition for the health professionals, as it provided a service to which women could be referred, something previously missing from care. The health professionals supported the lifestyle approach taken in the designed antenatal programme, as obesity is a multi-factorial problem. The health professionals felt that a group approach was needed as opposed to a one-to-one session. The health professionals all agreed that care should be provided in the community with hospital-based professionals involved in delivery. They felt an integrated services approach, in which hospital- and community- based health professionals and experts worked together to promote and deliver interventions for women with a BMI ≥30 kg/m^2^, would increase acceptance of the programme.

#### Continuous care that starts early

A lack of continuity of care made it difficult to provide obesity advice as no relationship was developed and there was limited opportunity for follow up. Obesity being discussed with women throughout their pregnancy was suggested as the best approach.

#### Impact on clinical practice

The impact of maternal obesity on health professionals was discussed in two ways; impact on time and role of resources. The health professionals felt that maternal obesity impacted on their time in two ways; there was no time in the appointment to address weight and caring for obese women took more time than usual due to providing extra information. In terms of resources, many health professionals discussed physical issues such as the risks of wrist and shoulder strain occurring when caring for these women. The need for suitable equipment to help health professions avoid injury when caring for women with a BMI ≥30 kg/m^2^ in terms of availability and training (e.g. slide sheets for moving and handling) were discussed.

### Theme 3 - Long-term impact of maternal obesity intervention

The health professionals viewed obesity as a wider public health issue and felt that addressing obesity as part of maternity care had the potential for a long-term impact on this public health issue. The health professionals viewed obesity as a continuous lifespan public health issue and felt that if women were educated during the antenatal stage about how to implement healthy lifestyle changes in their family, it may have a positive impact on the cyclic relationship of obesity. Finally, the importance of informing women of the possible long term physical consequences of being obese was discussed as many health professionals felt this was their responsibility and could impact on women’s long-term health decisions. The health professionals felt that they knew little about the possible effects of obesity on psychological well-being and reported offering women little psychological support.

## Discussion

The three themes will be discussed in terms of their relevance to the provision of maternity care services and interventions with a focus on the need for enhanced training for health professionals as a key component in the efficient care of women who have a BMI ≥30 kg/m^2^.

All of the health professionals felt that obesity was a public health issue and saw maternity care as having a key role to play in the long-term future of obesity prevention. This view is consistent with the literature that portrays obesity as a ‘global epidemic’ [30] and documents its association with life threatening conditions [30,31]. Providing lifestyle advice during maternity care was viewed by the health professionals as contributing to the wider public health issue of obesity in two ways. Firstly, providing education that could impact on the cyclical pattern of obesity in the family at the early stages of life and secondly, pregnancy is a good time to encourage long-term lifestyle changes (‘a window of opportunity’ as stated in a recent commentary [32] Pg.S50). These views are supported by a recent report that considers early interventions as the best way towards improvement of the lives of families [33] and existing literature that states pregnancy is a good time to intervene [34,35]. Research with women with a BMI ≥30 kg/m^2^ has found that although motivation to make change is apparent in pregnancy and advice is wanted in this stage, changes to weight are planned for the postnatal period [17,36-38]. Therefore, health professionals should endeavour to provide advice in the antenatal period that can also be applied to the postnatal period by women when they actively want to make changes to their lifestyles and weight. The antenatal lifestyle programme that was designed for the current feasibility study included information on the postnatal period to enable the women to actively make healthy lifestyle changes after the birth (e.g., examples of exercises that the women could do with their babies).

The barriers to discussing obesity, highlighted in this study, centred on communication (e.g., suitable wording), time and a lack of training. This finding resonates with existing evidence [17,18,37] and also provides information on the barriers to the implementation of the recently produced maternal obesity guidelines [9]. For example, the need for all health professionals providing maternity care to discuss obesity was reported as a way to combat the lack of continuity of care, something previously highlighted as a barrier to providing efficient care for women with a BMI ≥30 kg/m^2^[20]. Several practice points are evident, including the need for an antenatal lifestyle programme to be integrated into the maternal obesity care pathway. Having an antenatal lifestyle programme to which health professionals could refer women with a BMI ≥30 kg/m^2^ was supported by the health professionals as they felt that they did not personally have the time required or the skills or knowledge to address obesity with women. The health professionals felt that the antenatal lifestyle programme in the feasibility study was suitable as they supported the multi-component content (e.g., physical activity and diet), the community and the group setting. An integrated health care system such as this has been suggested to maintain long-term (physical and psychological) benefits of weight loss [32].

Several of the health professionals suggest that standardised obesity questions (similar to the Whooley questions for mental health [39]) within the maternity booking history or notes would make weight easier to approach (e.g., providing suitable questions). This is supported by existing literature where routine questioning has helped reduce the stigma attached to sensitive issues and increased health professionals’ confidence in discussing them [18,40-42]. Further research is required to design and validate efficient questions and to ensure their efficacy in clinical practice is evaluated.

The current findings suggest that health professionals feel that they need specific training in order to address obesity easily; the only relevant training that they reported receiving was breaking bad news. Tailored training to provide health professionals with the skills and knowledge to offer advice about healthy lifestyle choices to women with a BMI ≥30 kg/m^2^ is vital if maternal obesity guidelines are to be implemented effectively [19-23]. Our findings add to these studies by suggesting the content of this training; 1) increase knowledge of the possible risks associated with maternal obesity, 2) increase knowledge of suitable services to signpost women to for extra support and advice, 3) provide health professionals with the skills needed to approach the subject of weight and weight management in an acceptable and suitable manner and 4) provide information on the appropriate use of equipment for the management of maternal obesity. Training can be informed by studies such as this one and other exploratory studies [18]. Training should be delivered in two ways; through continuing professional development (CPD) and as an addition to the relevant University syllabuses (e.g., public health module). Increasing the knowledge of newly qualified health professionals may in turn increase their confidence and ability to discuss the issue of obesity.

The short length of the interviews reflected the busy lifestyles of the interviewees but resulted in rich and relevant data. There are several limitations with the current study. Firstly, the participants’ awareness of maternal obesity may have increased due to the visibility of the feasibility study. Secondly, this study was only conducted in two NHS hospitals in the North West of England with 30 health professionals so the results cannot be generalised to all health professionals delivering maternity care. Thirdly, other professionals have contact with women with a BMI ≥30 kg/m^2^ such as health care assistants and administrative staff. Their views were not sought and would need to be understood if we are to present a holistic picture of maternity care for women with a BMI ≥30 kg/m^2^. Finally, due to the exploratory nature of the study no demographics were collected although these may have an impact. For example, health professionals’ weight has been suggested as important if they are acting as role models [10]. Therefore, the possible influence of health professionals’ weight on the women’s perception of the obesity advice should be examined [43].

Several recommendations for health professionals when caring for women with a BMI ≥30 kg/m^2^ in clinical practice are suggested based on these findings. Firstly, the introduction of standardised questions to the maternity booking history or notes regarding weight and lifestyle should be explored. Secondly, health professionals require training to cover the following four areas: increase knowledge of the possible risks associated with maternal obesity; increase knowledge of suitable support services; provide them with the skills needed to approach the subject of weight and provide information on the appropriate use of equipment for the management of maternal obesity. Finally, specialist antenatal services and interventions to offer antenatal and postnatal advice to women with a BMI ≥30 kg/m^2^ are needed. The health professionals were in favour of the designed antenatal lifestyle intervention (TLC). These findings support the further examination of TLC to examine the effectiveness of this intervention for women with a BMI ≥30 kg/m^2^.

## Conclusion

The health professionals felt that providing lifestyle advice to women with a BMI ≥30 kg/m^2^ during maternity care may play a key role in addressing the public health issue of obesity. They felt women’s motivations were high during pregnancy so they may be more receptive to advice that has the potential for a long-term impact on them and their families. However, discussing maternal obesity was viewed as a challenge and the need for additional training was highlighted to enhance health professionals’ communication skills and increase their knowledge of obesity risks, support services and use of equipment to manage maternal obesity. Finally, further research is needed to explore appropriate antenatal and postnatal interventions to promote healthy lifestyles.

## Competing interests

The authors declare that they have no competing interests.

## Authors’ contributions

DMS was responsible for designing the study, conducted the interviews, analysed the transcripts and drafted the manuscript. AC analysed the transcripts. TL is principle investigator for the study and was responsible for the study design. All authors discussed and agreed on the content of the intervention and recruitment strategy. All authors read and commented on drafts of the manuscript and agreed on the final version.

## Pre-publication history

The pre-publication history for this paper can be accessed here:

http://www.biomedcentral.com/1471-2393/12/157/prepub

## References

[B1] Arrowsmith S Wray S Quenby S Maternal obesity and labour complications following induction of labour in prolonged pregnancy J Obstetricians Gynaecologists 2011 118 578 588 10.1111/j.1471-0528.2010.02889 PMC3085126 21265999

[B2] Denison FC Maternal obesity, length of gestation, risk of postdates pregnancy and spontaneous onset of labour at term J Obstetricians Gynaecologists 2008 115 720 725 10.1111/j.1471-0528.2008.01694.x PMC2344995 18410655

[B3] Watkins M Maternal obesity and risk for birth defects Pediatrics 2003 111 1152 1158 12728129

[B4] Denison FC Increased maternal BMI is associated with an increased risk of minor complications during pregnancy with consequent cost implications J Obstetricians Gynaecologists 2009 116 1467 1472 10.1111/j.1471-0528.2009.02222.x 19496775

[B5] Sebire NJ Maternal obesity and pregnancy outcome: a study of 287,213 pregnancies in London Int J Obes Relat Metab Disord 2001 25 1175 1182 10.1038/sj.ijo.0801670 11477502

[B6] Centre for Disease Control & Prevention Pregnancy nutrition surveillance. Nation. Summary of trends in maternal health indicators 2007 Available at: http://www.cdc.gov/PEDNSS/pnss_tables/html/pnss_national_table16.htm

[B7] Heslehurst N A nationally representative study of maternal obesity in England, UK: trends in incidence and demographic inequalities in 619323 births, 1989–2007 Int J Obes 2009 34 3 420 428 10.1038/ijo.2009.250 20029373

[B8] Centre for Maternal and Child Enquiries (CMACE) Maternal obesity in the UK: Findings from a national project 2010 London: CMACE

[B9] Centre for Maternal and Child Enquiries & Royal College of Obstetricians and Gynaecologists CMACE & RCOG Joint Guidance Management of women with obesity in pregnancy 2010 London: CMACE & RCOG

[B10] Cross-Government Obesity Unit Healthy weight, healthy lives: One year on 2008 Available at: http://www.dh.gov.uk/en/publichealth/healthimprovement/obesity/index.htm

[B11] National Institute for Health and Clinical Excellence (NICE) Improving the nutrition of pregnant and breast feeding mothers and children in low income households 2008 NICE, London: NICE Public Health Guidance 11

[B12] Campbell F Systematic review of dietary and/or physical activity interventions for weight management in pregnancy 2009 Sheffield: The University of Sheffield: ScHARR Public Health Collaboration Centre

[B13] Messina J Systematic review of weight management interventions after childbirth 2009 Sheffield: The University of Sheffield: ScHARR Public Health Collaboration Centre

[B14] Thangaratinam S Effects of interventions in pregnancy on maternal weight and obstetric outcomes: meta-analysis of randomised evidence Br Med J 2012 344 e2088 10.1136/bmj.e2088 22596383 PMC3355191

[B15] Tanentsapf I Heitmann BL Adegboye ARA Systematic review of clinical trials on dietary intervention to prevent excessive weight gain during pregnancy among normal weight, overweight and obese women BMC Pregnancy Childbirth 2011 11 81 10.1186/1471-2393-11-81 22029725 PMC3215955

[B16] Oteng-Ntim E Lifestyle interventions for overweight and obese pregnant women to improve pregnancy outcome: systematic review and meta-analysis BMC Med 2012 10 47 10.1186/1741-7015-10-47 22574949 PMC3355057

[B17] Smith D Lavender T The pregnancy experience for women with a body mass index >30 kg/m2; a meta-synthesis Br J Obstet Gynaecol 2011 118 779 789 10.1111/j.1471-0528.2011.02924.x 21385305

[B18] Lee D Haynes C Garrod D Exploring health promotion practice within maternity services 2010 Manchester: Stockport NHS Foundation Trust. Final report

[B19] Furness PJ Maternal obesity support services: a qualitative study of the perspectives of women and midwives BMC Pregnancy Childbirth 2012 11 69 21982306 10.1186/1471-2393-11-69 PMC3198957

[B20] Heslehurst N Obesity in pregnancy; a study of the impact of maternal obesity on NHS maternity services Br J Obstet Gynaecol 2007 114 334 342 10.1111/j.1471-0528.2006.01230.x 17261124

[B21] Davis DL Addressing obesity in pregnancy: The design and feasibility of an innovative intervention in NSW, Australia Women and Birth 2012 in press 10.1016/j.wombi.2011.08.008 21930449

[B22] Smith SA Community-based service provision for the prevention and management of maternal obesity in the North East of England: a qualitative study Public Health 2011 125 8 515 524 10.1016/j.puhe.2011.05.004 21794887

[B23] Olander EK The views of pre- and post-natal women and health professionals regarding gestational weight gain: an exploratory study Sex Reprod Healthc 2011 2 43 48 10.1016/j.srhc.2010.10.004 21147458

[B24] Salazar SS Assessment and management of the obese adult female: a clinical update for providers J Midwifery Womens Health 2006 51 3 202 207 10.1016/j.jmwh.2006.02.002 16647672

[B25] Medical Research Council Developing and evaluating complex interventions: new guidance 2008 Available at: http://www.mrc.ac.uk/Utilities/Documentrecord/index.htm?d=MRC004871

[B26] Smith D Study Protocol: the design of a community lifestyle programme to improve the physical and psychological well-being of pregnant women with a BMI of 30 kg/m2 or more BMC Public Health 2010 10 284 294 10.1186/1471-2458-10-284 20507580 PMC2887821

[B27] Department of Health Tackling Health Inequalities: 2004–06 data and policy update for the 2010 National Target 2007 London: Department of Health

[B28] NHS Information Centre Statistics on obesity, physical activity and diet: England 2011 2011 Leeds: The Health and Social Care information Centre

[B29] Braun V Clarke V Using thematic analysis in psychology Qual Res Psychol 2006 3 77 101 10.1191/1478088706qp063oa

[B30] World Health Organization Obesity. Preventing and managing the global epidemic. Report of a WHO consultation on obesity. WHO/NUT/NCD/981 1999 Geneva: WHO 11234459

[B31] Manson J A prospective study of obesity and risk of coronary heart disease in women N Engl J Med 1990 322 882 889 10.1056/NEJM199003293221303 2314422

[B32] Kapur A Pregnancy: A window of opportunity for improving current and future health Int J Gynecol Obstet 2011 115 Suppl. 1 S50 S51 10.1016/S0020-7292(11)60015-5 22099444

[B33] Allen G Early interventions; The next steps. An independent report to her Majesty’s Government 2011 Available at: http://www.childpovertysolutions.org.uk/UserFiles/file/GrahamAllenEarlyInterventionReport.pdf. Retrieved 7th April 2011

[B34] National Institute for Health and Clinical Excellence (NICE) Behaviour change at population, community and individual levels 2007 London: NICE Public Health Guidance NICE

[B35] Phelan S Pregnancy: a “teachable moment” for weight control and obesity prevention Am J Obstet Gynecol 2010 202 135.e1 e8 10.1016/j.ajog.2009.06.008 19683692 PMC2815033

[B36] McParlin C Objectively measured physical activity during pregnancy: a study in obese and overweight women BMC Pregnancy Childbirth 2010 10 76 84 10.1186/1471-2393-10-76 21114834 PMC3001702

[B37] Weir Z Physical activity in pregnancy: a qualitative study of the beliefs of overweight and obese pregnant women BMC Pregnancy Childbirth 2010 10 18 24 10.1186/1471-2393-10-18 20426815 PMC2879230

[B38] Aviram A Hod M Yogev Y Maternal obesity: Implications for pregnancy outcome and long-term risks – a link to maternal nutrition Int J Gynecol Obstet 2011 115 Suppl. 1 S6 S10 10.1016/S0020-7292(11)60004-0 22099446

[B39] Whooley M Case-finding instruments for depression. Two questions are as good as many J Gen Intern Med 1997 12 439 445 10.1046/j.1525-1497.1997.00076.x 9229283 PMC1497134

[B40] Price S Baird K Salmon D Does routine antenatal enquiry lead to an increased rate of disclosure of domestic abuse? Findings from the Bristol Pregnancy and Domestic Violence Programme Evidence Based Midwifery 2007 5 100 106

[B41] Ratnapalan S Universal HIV testing in pregnancy Can Family Physician 1999 46 508 410 PMC2144963 10751987

[B42] Sherr L Bergenstrom A Hudson N Consent and antenatal HIV testing: the limits of choice and issues of consent in HIV and AIDS AIDS Care 2010 12 307 312 10.1080/09540120050042963 10928208

[B43] Department of Health Healthy Weight, Healthy Lives. Resources for Health Professionals Tool E6 2009 Available at: http://www.fph.org.uk/uploads/HealthyWeight_SectE.pdf. Retrieved: 30th August 2011

